# Home Department

**Published:** 1886-08

**Authors:** 


### HOME DEPARTMENT.

**Flies.**—The greatest summer pests in most houses are flies. This may be largely prevented if not wholly obviated in different ways, the neatest of which is to screen the windows against their intrusion, but in most cases, this is found to be too troublesome or expensive. Fly papers for destroying flies are of two kinds, a sticky substance is smeared over the surface of paper and exposed in some suitable place. This coating may be made of birdlime with a mixture of molasses, but it is objectionable as causing a lingering and cruel death. Soft thick paper, soaked in a preparation of sugar, water and arsenic, and when in use, kept moist is usually preferred.

**Egg Hatching.**—Automatic incubators for hatching eggs are coming very generally into use. The later inventions in this line have acquired great perfection, leaving very little to be done by the operator, beyond keeping the vitalizing lamp at a steady flame, and attending to the proper disposal of the little strangers, when they first emerge from their white-rowed cells. Of late artificial hatching has become quite a fashionable amusement with our suburban gentlemen and ladies, who do not seem to suffer any qualms of conscience in setting aside their natural mother and overruning their grounds with any number of unbrooded orphans. The art is by no means new, for we are made aware that it was practiced by the ancient Egyptians, and revived in Western Europe as early as 1775. All that is required is a continuous, even slightly moist temperature of one hundred degrees fahrenheit. The hatching requires twenty-one days, and the chickens, as fast as hatched, should be removed to a suitable enclosure of like temperature, which is gradually lowered until the open air is reached. Crushed millet seed is considered the best food for a day or two, which the little family will pick up for themselves from their first endeavors at gaining a livelihood.

**Home Washing.**—The advantage of home washing is that it can be superintended by a member of the family, who will see that it is properly done, and no deleterious cleansing substances allowed to be used in the process. Since the introduction of Chinese laundrymen into our larger towns and cities, who have come to largely monopolise this branch of industry, it is almost impossible to procure from them a washing in the regular way that does not show the free use of chloride of lime, or some equally destructive agent. It requires only a few such washings to render garments valueless. Yet there is scarcely a Chinaman in the business who is frank enough to admit that he makes use of any like injurious substance.

Washing woolens in very hot water makes them hard. To secure excellence, pour into cold water an equal quantity of boiling soap suds, made with two or three ounces of good soap to the gallon, well dissolved and boiled. In this water thus prepared the woolens, after being previously soaked, should be immediately washed, without hard rubbing, which injures the fabric. Rinse in abundance of warm water, wring quickly and dry in the open air. This is essential with woolens, as drying indoors, especially by a fire, hardens them.

**Children's Milk.**—Keep the child's milk separate from that intended for the rest of the household. Have ready a pan that has been properly cleansed by being thoroughly washed and rinsed, and rinsed again in a solution of bicarbonate of soda, to receive the milk. In hot weather the milk should be placed on the fire and the temperature be brought to a point just short of boiling—do not let it boil. It should then be put in a proper receptacle and placed in a cool place, or a refrigerator which does not contain vegetables. It should then be prepared as follows: Milk, one-half pint; pure water, one-half pint; powdered sugar of milk,' one tablespoonful; phosphate of lime, one grain. Dissolve the sugar and lime in the water and add the milk. This is the nearest approach to human milk that can be prepared. As the child grows, add less water. A good rule is this: Until one month add two-thirds water; one-half water until three months, one-third water until the sixth month, one-fourth until the ninth month and one-sixth until one year of age, when the child can take the milk clear, and often in combination with some of the infant food upon the market. The water should be boiled and allowed to cool before using, as this will purify it by destroying any germs it may contain. Some will no doubt ask why heat the milk. During high temperature, when the mercury ranges from 86 to 100 degrees, there is rapid decomposition of milk, the caseine is rapidly coagulated, and in this condition it exerts a peculiar action (catalytic) upon the other solids of the milk, particularly upon the sugar, forming lactic acid. The milk loses its alkaline reaction and becomes sour, when it is not fit for further use. The heating of the milk retards this process.—*The Anti-Adulteration Journal, August,* 1886.

---

**A tramp** asked for something to eat at a restaurant in Mattoon, Ill. The cook offered to him two dozen fried eggs if he could eat them all. The tramp agreed and the eggs were set before him. After having eaten twenty-one eggs, a loaf of bread and some sardines, he fell asleep.

